# A set of genes previously implicated in the hypoxia response might be an important modulator in the rat ear tissue response to mechanical stretch

**DOI:** 10.1186/1471-2164-8-430

**Published:** 2007-11-23

**Authors:** Vishal Saxena, Dennis Orgill, Isaac Kohane

**Affiliations:** 1 Surgery, Brigham and Women's Hospital, Boston, MA, USA; 2 Harvard Medical School, Boston, MA, USA; 3 Children's Hospital Informatics Program, Children's Hospital, Boston, MA, USA; 4 HST, Massachusetts Institute of Technology, Cambridge, MA, USA

## Abstract

**Background:**

Wounds are increasingly important in our aging societies. Pathologies such as diabetes predispose patients to chronic wounds that can cause pain, infection, and amputation. The vacuum assisted closure device shows remarkable outcomes in wound healing. Its mechanism of action is unclear despite several hypotheses advanced. We previously hypothesized that micromechanical forces can heal wounds. To understand better the biological response of soft tissue to forces, rat ears in vivo were stretched and their gene expression patterns over time obtained. The absolute enrichment (AE) algorithm that obtains a combined up and down regulated picture of the expression analysis was implemented.

**Results:**

With the use of AE, the hypoxia gene set was the most important at a highly significant level. A co-expression network analysis showed that important co-regulated members of the hypoxia pathway include a glucose transporter (slc2a8), heme oxygenase, and nitric oxide synthase2 among others.

**Conclusion:**

It appears that the hypoxia pathway may be an important modulator of response of soft tissue to forces. This finding gives us insights not only into the underlying biology, but also into clinical interventions that could be designed to mimic within wounded tissue the effects of forces without all the negative effects that forces themselves create.

## Background

### Clinical context

**Figure 1:**
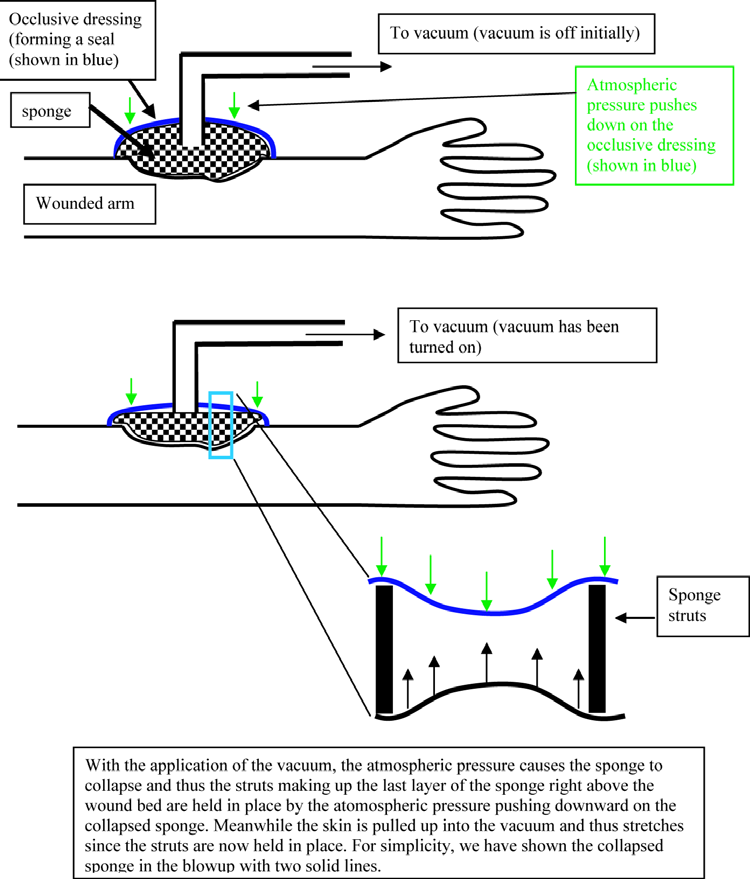
Vacuum application. The upper part of the figure has the vacuum off. The sponge is therefore uncollapsed. The lower part of the figure has the vacuum on. The sponge has collapsed. The blowup shows how the skin stretches once the vacuum is applied.

Worldwide, wounds pose a major health issue. Lower extremity ulcers alone cost the US Medicare system $1.5 billion. Unless wound therapies see a large improvement, we will see escalating treatment costs and morbidity as the population ages and as the incidence of diabetes and obesity increases. An understanding of the mechanisms underlying wound healing will shed light on how normal physiology adapts to changes in the normal homeostatic environment. The vacuum assisted closure (VAC) device (KCI, San Antonio Texas) is a relatively new modality in wound healing. Although the device has been shown to accelerate wound healing, its mode of action remains to be proven convincingly. Figure 1 shows the application of the device. This involves packing a polyurethane sponge into the wound bed and then sealing the wound including the sponge with an occlusive dressing that has one outlet tube going to a vacuum (a vacuum of about 115 mmHg is applied through the tube). Theories about how this device obtains its efficacy range from a reduction in bacterial load [1] to a reduction in edema. The blowup in Figure 1 shows that with the application of the vacuum the skin is pulled into the intra-strut spacings of the sponge thereby stretching the skin.

**Figure 2:**
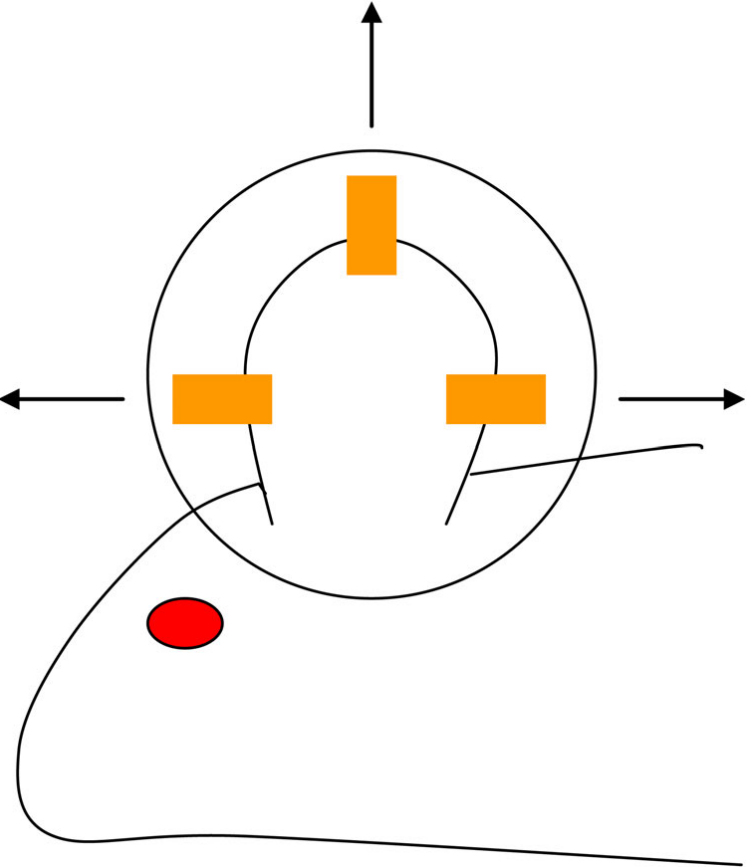
rat force apparatus. Strips of latex (shown in orange) are pulled by rubber bands (not shown) in three directions.

In an earlier publication, we have used a numerical model to show how the imposition of the vacuum forces the tissue to stretch by small amounts (micromechanically) as the tissue is 'pulled' into the empty space defined by regular holes in the sponge causing a tension to be applied to the tissue substrate and through it to the cells embedded within the substrate [2]. Previous work [3] by Donald Ingber, one of the co-authors on our microstreatch paper [2] has shown that cells stretched in vitro proliferate, whereas cells that are not stretched are cell cycle arrested. We thus, in that paper, hypothesized that through a similar mechanism the cells in the wound bed subjected to stretch by the application of negative suction pressure are also proliferating and thus helping the wound heal faster [2]. If we are able to understand the molecular mechanisms that underlie tissue response to forces, then we could substitute forces with interventions that mimic these molecular events. The effects of tissue expansion mechanisms [4] are little studied in the literature. A rat ear was chosen as the system in which to study the effects of forces on tissues in a perfused system (Figure 2). The rat ear is thin and the blood vessels can be easily visualized under a light microscope. Further, it is highly amenable to force experiments in vivo.

## Results

### Enrichment analyses

We ran the absolute enrichment, the upregulated enrichment (the standard gene set enrichment analysis or GSEA), and the downregulated enrichment analyses on our dataset as explained in the methods section. The results are presented in Tables 1 through 3 respectively. The absolute enrichment analysis produced the hypoxia gene set as the top ranked gene set of the 173 gene sets tested. Also, 'response to mechanical stimulus' achieves the second highest rank. The members of the hypoxia gene set are listed in Table 4 along with their paired t scores. Positive t scores (shown in blue) indicate overall upregulation over the 8 time points, while negative scores (shown in red) indicate overall downregulation over the 8 time points.

**Table 1:** Absolute enrichment

Top gene set	EASE GO categories (for some gene sets)	P value
hypoxia		0.0033
response_to_mechanical_stimulus		0.015
c3_U133_probes	RNA binding (MF), ribonucleoprotein complex (CC), cytosolic ribosome (CC), structural constituent of ribosome (MF), ribosome (CC), small ribosomal subunit (CC), cytosolic small ribosomal subunit (CC), eukaryotic 48S initiation complex (CC), eukaryotic 43S preinitiation complex (CC), nucleic acid binding (MF)	0.018
MAP00340_Histidine_metabolism		0.004
c27_U133_probes	Intracellular (CC), regulation of translation (BP), nucleic acid binding (MF), RNA binding (MF), cell cycle (BP), DNA metabolism (BP), response to DNA damage stimulus (BP), response to endogenous stimulus (BP), DNA repair (BP), regulation of translational initiation (BP)	0.016
MAP00280_Valine_leucine_and_isoleucine_degradation		0.0096
c23_U133_probes	Oxidoreductase activity (MF), microbody (CC), peroxisome (CC), transporter activity (MF), ethanol metabolism (BP), ethanol oxidation (BP), catalytic activity (MF), cytoplasm (CC), carboxylic acid metabolism (BP), organic acid metabolism (BP)	0.012
c6_U133_probes	Epidermal differentiation (BP), Ectoderm development (BP), histogenesis (BP), intermediate filament cytoskeleton (CC), Intermediate filament (CC), morphogenesis (BP), organogenesis (BP), structural molecule activity (MF), development (BP), cytoskeleton (CC)	0.014
mitochondr_HG-U133A_probes		0.008
MAP00140_C21_Steroid_hormone_metabolism		0.0027

**Table 2:** Up regulated analysis

Gene set	EASE GO categories	P value
response_to_mechanical_stimulus		0.085
c26_U133_probes	Sexual reproduction (BP), reproduction (BP), spermatogenesis (BP), male gamete generation (BP), intracellular (CC), gametogeneis (BP), chaperone activity (MF), M phase (BP), nuclear division (BP), spindle (CC)	0.014
c6_U133_probes	Epidermal differentiation (BP), Ectoderm development (BP), histogenesis (BP), intermediate filament cytoskeleton (CC), Intermediate filament (CC), morphogenesis (BP), organogenesis (BP), structural molecule activity (MF), development (BP), cytoskeleton (CC)	0.023
c3_U133_probes	RNA binding (MF), ribonucleoprotein complex (CC), cytosolic ribosome (CC), structural constituent of ribosome (MF), ribosome (CC), small ribosomal subunit (CC), cytosolic small ribosomal subunit (CC), eukaryotic 48S initiation complex (CC), eukaryotic 43S preinitiation complex (CC), nucleic acid binding (MF)	0.003
Hum_Fb_Serum_EarlyTF		0
OXPHOS_HG-U133A_probes		0.131
c27_U133_probes	Intracellular (CC), regulation of translation (BP), nucleic acid binding (MF), RNA binding (MF), cell cycle (BP), DNA metabolism (BP), response to DNA damage stimulus (BP), response to endogenous stimulus (BP), DNA repair (BP), regulation of translational initiation (BP)	0
mitochondr_HG-U133A_probes		0.018
MAP00710_Carbon_fixation		0.004
c14_U133_probes		0.002

**Table 3:** Down regulated analysis

Gene set	P value
MAP00280_Valine_leucine_and_isoleucine_degradation	0.008
MAP00340_Histidine_metabolism	0.005
MAP00310_Lysine_degradation	0
MAP00380_Tryptophan_metabolism	0.002
MAP00632_Benzoate_degradation	0.01
hypoxia	0
MAP00562_Inositol_phosphate_metabolism	0.004
MAP00052_Galactose_metabolism	0
MAP00521_Streptomycin_biosynthesis	0.001
cluster7_LPS_mouse_urinary.txt	0

**Table 4:** Hypoxia geneset details

Hypoxia Probe Set ID	Gene symbol (or title)	t score
1368286_at	Slc2a8	2.29
1370080_at	Hmox1	2.03
1381936_at	Camk2g	1.48
1371719_at	Brd2	1.17
1371289_at	unknown	-0.44
1375650_at	Brd4	-0.51
1368322_at	Sod3	-1.07
1369703_at	Epas1	-1.19
1387605_at	Casp12	-1.19
1387818_at	Casp4	-1.26
1387667_at	Nos2	-1.52
1373916_at	Ep300	-1.65
1369186_at	Casp1	-1.67
1374863_at	Similar to retinoid binding protein 7 (predicted)	-1.73
1369307_at	Ep300	-2.11

It should be noted that in gene set enrichment analysis we typically do not calculate the P values for each gene separately. Rather we perform the permutation test (as we explain in the methods section) and look for the P values of the gene set. This is why we have not listed the P values of the genes in the hypoxia pathway. In the upregulated enrichment analysis, 'response to mechanical stimulus' achieves the top position. Interestingly, the c26 cluster which is enriched for Reproduction genes from EASE and which was derived from Mootha's original gene sets has obtained second rank in the up regulated analysis. There are a total of 36 c clusters derived by Mootha through the use of self organizing maps over the GNF mouse expression atlas which itself is derived from expression profiling of different mouse tissue [5]. There were no gene sets in the down regulated analysis that had an ambiguous interpretation and thus none had to be analyzed through EASE. The Hypoxia gene set obtains rank 6 in the down regulated enrichment analysis. To see how members of the hypoxia gene set may be co-regulated, we ran a co-expression network analysis.

### Co-expression network analysis

**Figure 3:**
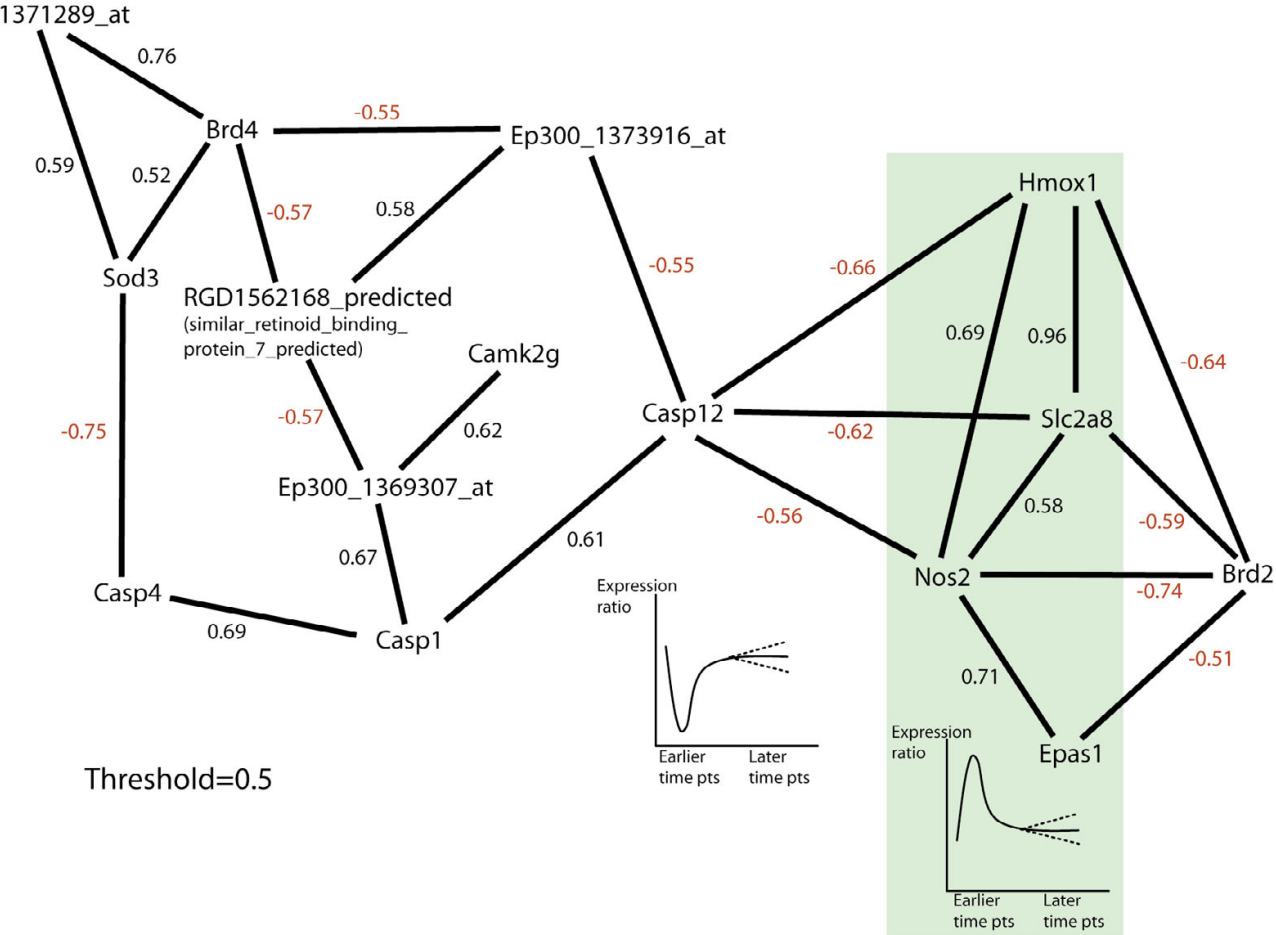
Co-expression network. NOS2, Hmox1, slc2a8, and Epas1 all show a rise in the early time points while Casp12 (and genes positively correlated with it) show an early drop. The figures underneath these genes show this time trajectory with dashed lines showing that the later time points do not necessarily show a flat response. The correlations between genes are shown next to the lines connecting genes.

Pairwise correlations between the gene expression trajectories provide the correlation network weights shown in Table 5. The correlation network structure based on a threshold of 0.5 is shown in Figure 3. To obtain the connectivity values (Table 5), absolute values of the correlations were summed for each gene.

**Table 5:** Co-expression network analysis

Gene symbol (or probeset when symbol unavailable)	Connectivity values (in descending order)
Nos2	5.6852
Casp12	5.365
Casp4	5.3197
Brd4	5.038
Brd2	4.8786
Ep300_1369307_at	4.7816
Casp1	4.7395
Hmox1	4.6694
Slc2a8	4.5987
Sod3	4.5886
RGD1562168_predicted	4.3523
Epas1	4.1956
1371289_at	4.1945
Ep300_1373916_at	3.8843
Camk2g	2.888

### Expression ratio plot over time

**Figure 4:**
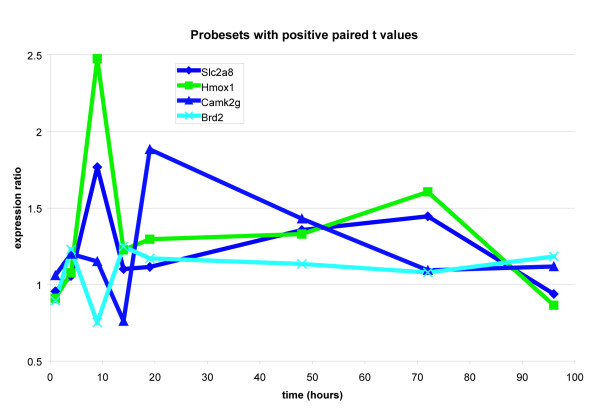
The trajectories of the genes that showed an overall positive paired t score. Not all time points however are greater than one (when we compare genes by taking differences, upregulated genes are positive, while when we compare genes by taking ratios, upregulated genes show expression ratios greater than one) as we see for example with Camk2g.

The hypoxia gene set members were further visualized by plotting the stretch to control expression values in three groups. The first group was made up of the positively upregulated members of the hypoxia pathway (positive given by the sign of the paired t score). The second and third groups were obtained by taking the remaining members of the hypoxia pathway (these all had negative paired t scores) and then splitting them by inspection of their time trajectories into two groups. These results are presented in Figures 4 through 6.

**Figure 5:**
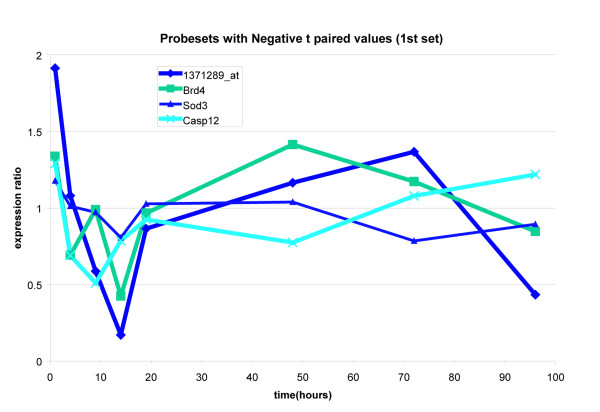
The first set of genes that show an overall negative paired t score. All these genes show a dip at the earlier time points in the stretch to control expression ratios.

**Figure 6:**
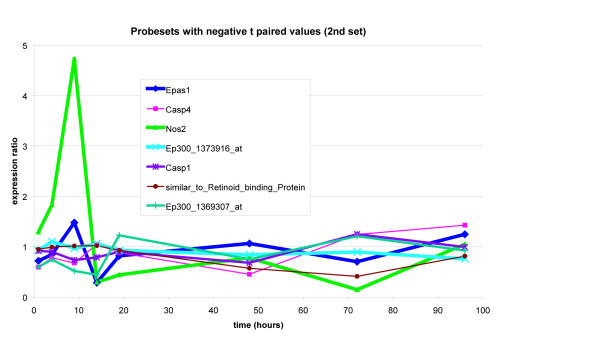
This is the second set of genes that show an overall negative paired t score. Here again we note that not all time points display less than one expression value ratios. For example, NOS2 shows a very large rise at the earlier time points.

The time trajectories of the members of the hypoxia gene set show two prototypical behaviors. They show either a sharp spike at earlier time points that is then dampened or they show a sharp dip at earlier time points that then recovers at later time points. These two are displayed on the co-expression network graph (Figure 3). We note that the members shown in light green shading show the first prototypical behavior while members negatively correlated with this set show the dip behavior (Casp12 for example). Ep300_1373916_at and RGD_1561628_predicted are negatively correlated with Casp12 and thus show a rise at earlier time points but we haven't shaded these because their rise isn't as pronounced.

## Discussion

The response to mechanical stimulus' gene set was ranked at number two in our absolute enrichment analysis and it was ranked at number one in our upregulated enrichment analysis. This gives us a strong measure of confidence that our t paired statistic is capturing relevant themes in our rat ear stretch system.

In the absolute enrichment framework, Permutation testing [5] gave the hypoxia gene set a highly significant P value of 0.0033 at the 0.05 level. The two prototypical behaviors seen in the time trajectories (seen in Figures 4 through 6) tell us that the genes in our hypoxia pathway show either a sharp rise or a sharp fall at earlier time points and then recover. This behavior was then compared with what has been reported in the literature. Specifically, we note that SOD3 shows a decline at earlier time points and then a recovery while NOS2, Slc2a8, and Hmox1 all show sharp rises at earlier time points. Maiti et al [6] have stated that "under hypoxic stress, the cellular defence systems such as antioxidant enzymes (GPx, GR, SOD, etc.) get disturbed and their activity decreases." Further, they report that in the rat brain, hypoxia leads to an increase in nitric oxide. In our system, NOS2 is sharply rising at earlier time points while SOD3 is falling at earlier time points.

It has also been reported in the literature that NO production upregulates heme oxygenase (Hmox-1) production [7,8]. This may explain why we see a sharp rise in Hmox in the early time points. It also may explain why on our co-expression network graph, NOS2 and Hmox show a positive correlation of 0.69. At later time points, however, NOS2 and Hmox-1 do not move together (for example NOS2 shows downregulation while Hmox shows upregulation). Nitric oxide isn't the only mediator of Hmox-1 upregulation. For example, it has been reported that Hmox-1 is the major stress protein induced by UVA, hydrogen peroxide and arsenite [9]. Further, it is known that Hmox-1 expression is linked to tissue stretch. Many pathways lead to heme oxygenase I expression through renal injury [7]. Mechanical stress has been shown to cause oxidant stress [10] and Hmox-1 levels are increased when cells are exposed to oxidative stress [11]. Hmox-1 prevents oxidant-induced microvascular leukocyte adhesion [12,13]. Hmox-1 has cytoprotective roles [14], and is anti-inflammatory [15].

Hmox-1 itself is a negative regulator of NO [16,17]. Thus, if Hmox-1 is upregulated independently of NO (as may be happening at the later time points), then NO is strongly inhibited. For example, hemin can upregulate Hmox-1 (independently of NO). This induction has been shown to strongly inhibit NO production of LPS-activated macrophages [18,19]. Loike *et al.*[20] have reported that hypoxia leads to glucose transporter expression in endothelial cells. Thus, it may be that we see a sharp spike in slc2a8 (which is a glucose transporter [21,22]) as a consequence of hypoxia at earlier time points in our system.

Thus, our system may be increasing glucose intake to make up for lack of oxygen, and it seems that our system may be undergoing hypoxia [23,24] at least at earlier time points. According to [23], "...the molecular mechanisms by which muscle contraction/hypoxia increase glucose uptake are less well defined, although they appear to be independent of the PI3K pathway. Most intriguing is the observation that the recently identified hormone adiponectin also stimulates skeletal muscle glucose uptake in a PI3K-independent manner." The adiponectin gene is not part of the hypoxia gene set. We went to our dataset to see if seemed to show a trend similar to the genes in our hypoxia pathway. The adiponectin gene showed an upregulation or a rise at earlier time points dropping to a downregulation at later time points (plot not shown).

To our knowledge this is the first evidence that tissue stretch may lead to hypoxia. However, we should stress that we do not have replicates at each time point. Thus, the conclusions on the time trajectories follow from the results of the enrichment analysis and not the other way round. Because we feel that the hypoxia pathway is important, we then study its time trajectory in more detail for further insights.

## Conclusion

Our results show that the hypoxia pathway is clearly the most important in this dataset. This finding gives us insights not only into the biology that underlies the tissue response to forces, but also into clinical interventions that could be designed to mimic within wounded tissue the effects of forces without all the negative effects that forces themselves create (wound separation, pain and so on). Mimicking the effects of forces through the excitation of important parts of our hypoxia pathway through the use of interference RNA or other gene therapy interventions could make an important impact on the suffering that delayed wound healing creates in various disorders, primary among them being diabetes.

## Methods

### Applying forces to rat ears and obtaining the ear RNA

Male wistar rats weighing approximately 180 g were chosen for this study. All animal studies were conducted according to institutional guidelines. Strips of latex were fastened to the ears and, using appropriately calibrated rubber bands, forces measuring 50 g were applied to the ears at three points (Figure 2). Eight time points were sampled at 1 h, 4 h, 9 h, 13 h, 19 h, 48 h, 72 h, 96 h. Three rats were used per time point. One ear of each rat (chosen randomly) was stretched while the other served as control. At the time points listed above, the ears were excised and immediately frozen in liquid nitrogen and then stored at -80°C. The three stretched ears at each time point and the three unstretched ears at the same point were separately pooled (70 to 80% of the whole ear was used) and the RNA extractions carried out. The RNA was then hybridized to RAE 230 2.0 Affymetrix rat gene chips. MAS5 was used to obtain the expression values from the images. Expression datasets will be deposited at geodatasets.

### Analysis scheme

To gain insights into the molecular mechanisms and pathways underlying the cellular response to forces and tissue stretch, a gene expression analysis was conducted by stretching rat ears (see Figure 2) over a period of five days and then obtaining their expression profiles using Affymetrix arrays. Traditional techniques that analyze gene expression datasets rely on clustering techniques that do not take advantage of pre-existing information about pathways thus not taking account of biological context. This is also true of other analyses that rank genes by fold change or other measures of significance. Enrichment techniques which include The Absolute Enrichment (AE), The Gene Set Enrichment Analysis (GSEA), and what we call The Down Regulated Analysis (DRE) are powerful analysis techniques that not only take advantage of pre-existing pathway knowledge but also in their implementation increase the signal to noise ratio thereby giving us a higher chance of capturing subtle signals in the expression set. Enrichment techniques start by using a ranking metric to reorder the expression dataset.

Next, a priori created groups of genes are scored on this reordered dataset and ranked from most highly scored at the top of the reordered dataset to the least highly scored or 'enriched' groups or gene sets. Thus, rather than looking at significance of each gene within the expression dataset, we look at significance of groups of genes. Since the gene set enrichment analysis is not limited by the type of statistic used, we should use a statistic that will be best at elucidating the changes between the groups that we are studying. We thus conducted our analysis by reordering our dataset using the paired t-statistic as our ranking metric (as our dataset consists of paired data points and thus this statistic will be best for capturing differences). Enrichment techniques can be powerful tools in analyzing data. However, within the enrichment analysis framework, people have focused solely on upregulation. They have perhaps neglected to look at down regulation. Downregulation itself can be just as important as upregulation [5]. However, both downregulated and upregulated (enrichment) analyses may miss differential effects that show some balance between up and downregulation (what essentially we have termed "homeostatic" systems) [5].

**Figure 7:**
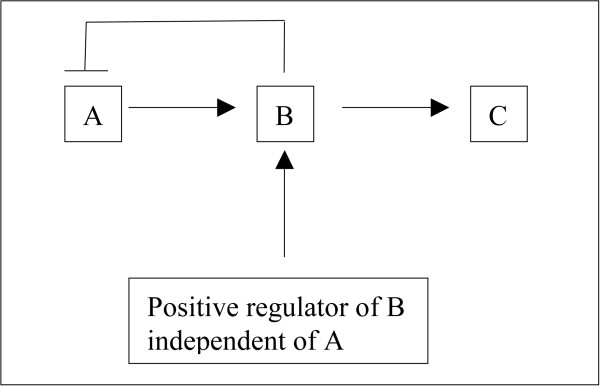
A is downregulated when B is upregulated independently of A.

**Figure 8:**
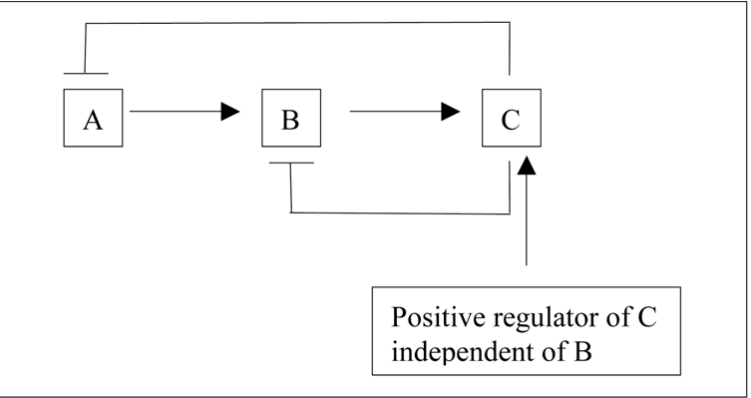
A and B both are downregulated when C is upregulated independently of B.

**Figure 9:**
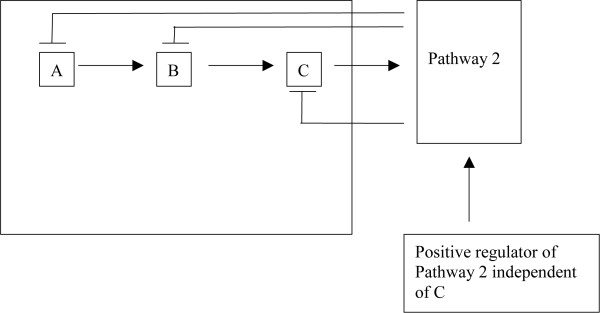
The whole pathway can be downregulated when a downstream pathway (pathway 2) is upregulated independently of it.

**Figure 10:**
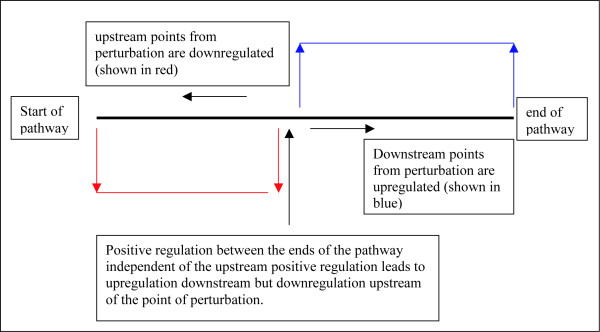
The pathway is shown by the thick horizontal black line (individual parts of the pathway are not depicted). The upward pointing black line with arrow shows an input that positively regulates a component towards the middle of the pathway upregulating the downstream parts of the pathway while leading to downregulation of the upstream components (as explained in Figs A through C above).

Let's look at a (metabolic) pathway (with feedback) to make this more clear. Let's say a pathway has 3 members A through C, and let's say A leads to upregulation of B which leads to upregulation of C (Figure 7). Further, if B can be upregulated independently of A, then B will downregulate A and if C can be upregulated independently of B, then its upregulation will downregulate both A and B (Figure 8). And further, if C itself feeds into another pathway which itself (the pathway) may be upregulated independently of C, then that pathway when independently upregulated will downregulate A, B, and C (Figure 9). We can essentially perturb a pathway at any point from start to finish (and not just at the start or at the end). If we perturb it in between such that there is a good mixture of both upregulation (coming from the downstream parts of the pathway) and downregulation (coming from the upstream parts of the pathway through feedback mechanisms), then this pathway may be captured by the absolute enrichment analysis (see Figure 10). For more details on absolute enrichment see below as well as ref [5]. (It must be noted that pathways don't have to have this serial relationship as in our conceptualized pathway. There can be bifurcations and so on.)

In essence, we are really looking for a change between an "affected" condition and a "nonaffected" or control condition. A change can be either an upward change (upregulation) or a downward change (downregulation). Thus, we're really looking for the highest differential regulation [5]. Sometimes, the absolute enrichment technique captures gene sets that are seen (at the very top) in either the up or down regulated analyses. Many times, however, it captures other gene sets. Because we run an exhaustive search through enrichment techniques that encompasses both up (or down) regulated and absolute enrichment techniques, we test whether the absolute enrichment technique captures anything extra at the top (of the list of ranked gene sets) compared with either of the up or downregulated techniques.

When what the absolute enrichment captures is found at the top of the up or downregulated analyses, then this analysis doesn't add new information (although a few of the rest of the top ranked gene sets in all the enrichment analyses can still be useful to study). However, when it does capture something new, we focus on it (or at least look at its result) because what it has captured is the most differentially expressed [5]. In our analysis, we captured a new gene set that was higher ranked than even the highest ranked upregulated and downregulated gene sets. Thus, we studied this gene set in more detail. This included running a co-expression network analysis (the fact that we *could *even run this analysis was driven by the small size of the top ranked hypoxia gene set).

Thus, to recap, our analysis scheme consisted of choosing the appropriate ranking metric (or statistic), running the three enrichment techniques, and then focusing on the gene set obtained from the absolute enrichment analysis (since it captured the most differentially regulated gene set). Our results show that the response to hypoxia pathway was the most differentially regulated pathway and that this pathway showed a homeostatic (comprising components of upregulation and downregulation) response. We believe that this pathway is being most differentially expressed through a homeostatic perturbation.

The rationale for the AE is that in a time series analysis it may be important to see how the system responds to the (mechanical stretch) perturbations that we have imposed on the system by regulating itself. Pathways are essentially composed of elements that respond in tandem to perturbations. Often these responses are self controlled through feedback mechanisms and thus homeostatic response analyses can often give us powerful indicators of pathways that are important in gene expression datasets.

### Enrichment analyses

**Figure 11:**
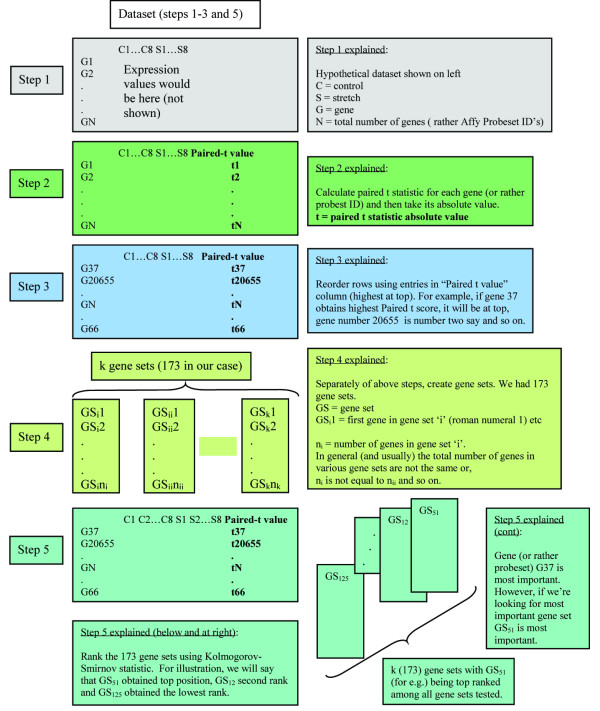
Steps in conducting enrichment analyses using absolute enrichment as an example.

Our data analysis uses enrichment techniques to understand important pathways in our system. Enrichment techniques start out by reordering the dataset using an appropriate statistic (please see Figure 11 for an overview of these techniques using absolute enrichment as an example). Consider the dataset to be a rectangular two dimensional array with the different conditions (control or stretch) represented by columns and the different genes represented along the rows. A suitable statistic is chosen that best helps us differentiate between stretch and control for each gene. Mootha's implementation of the gene set enrichment analysis (GSEA) [25] used the signal to noise ratio statistic to differentiate between the affected condition in his dataset and the control condition. Our dataset has paired samples (in the sense that one ear of the same rat was subjected to stretch while the other ear from the same rat at the same time point served as control). Thus, a better statistic to use in our case is the paired t-statistic since it takes better account of pair-wise differences. Enrichment techniques are not limited to the type of statistic used as the ranking metric. Any statistic that best lets us differentiate between the two classes (control versus stretch) can be used [5] (please see the Appendix for a description of the paired t-statistic).

Once the ranking metric is calculated, the dataset is reordered (in our case, we used the paired t statistic). This is where the three enrichment techniques that we used create different orderings. The GSEA gives us an ordering from most upregulated to the least upregulated. The downregulated enrichment or DRE ranks from most downregulated to the least downregulated. The absolute enrichment [5] uses the absolute value of the ranking metric (the paired t-statistic in our analysis) and then reorders the dataset. Whereas both the standard GSEA [25] and the DRE look for unidirectional regulations, the AE looks at a combined regulation and therefore is more effective at picking homeostatic systems. The absolute enrichment (AE) technique [5] looks at homeostatic perturbations rather than sole up or down regulations. Because it takes absolute values of the genes and then enriches gene sets on this absolute value ranked dataset, the AE becomes less sensitive to less perturbed genes. Once the dataset is reordered, we then test various a priori created gene sets to see how important each is vis-à-vis the reordering (construction of gene sets is explained in the next sub section). In contrast to strategies that look purely for most important genes, we are now looking for significant groups of genes or gene sets. When we look at a single gene, we look for how far up on a reordered dataset (reordered say by fold change) the gene finds itself. In an enrichment technique, however, we look for how far up the gene set finds itself in the reordered dataset. The measure of how far 'up' a gene set is placed on the reordered dataset is given by the enrichment score (ES). The enrichment score is calculated in the following way. Calculate the Kolmogorov Smirnov statistic for each probeset ID in our dataset given by,

$$XN=-\sqrt{\frac{G}{N-G}}$$

if the probeset ID is not part of the gene set and by,

$$X=-\sqrt{\frac{N-G}{G}}$$

if the probeset ID is part of the gene set, where G is the number of genes in the gene set and N is the total number of genes (or rather probeset IDs in the whole dataset – we had 31099 probeset IDs).

We next compute a running sum of this statistic on the reordered dataset. Where the running sum reaches a maximum score is our enrichment score for the gene set in question [25,5]. (Figure 11 shows the overall steps in running enrichment techniques). The gene sets are then ranked from highest enrichment score to the lowest. To test whether the results of the analysis are significant, we perform permutation testing of the dataset. To obtain the permutes, we swapped random stretch samples with random control samples. One thousand permuted datasets were constructed. All three enrichment analyses were run on each of these newly constructed datasets, giving us one thousand rankings of gene sets based on their enrichment scores (for each enrichment analysis). We then counted the number of times each gene set achieved top rank in the one thousand permutes. This number divided by 1000 gave us the permutation test P value. If this number was lower than 0.05, then the gene set was significant. For more details on the permutation test, please see Mootha *et al.*[25]. We didn't run technical replicates at each time point because our analysis is looking at an aggregate change between the stretched ear and control ear conditions across all time points. By doing this we look for overall changes between the eight time points versus their corresponding paired controls. We next describe how the various gene sets that were used in the enrichment techniques were constructed.

### Construction of gene sets

Many gene sets were compiled by the use of rat orthologs of previously published genesets [25] (according to [26] "it is estimated that 90% of rat genes have orthologs in the mouse and human genomes that have persisted since they shared a common ancestor"). Gene sets are essentially collections of genes. To derive additional gene sets that may show relevance, we searched the literature for different analyses that have been performed on cell excitation such as response to serum. When these analyses listed groups of genes obtained through various clustering or classifying techniques, we created gene sets for the various clusters listed in the papers. Other gene sets were created by looking up all possible relevant keywords at the geneontology.org website (angiogenesis, response to mechanical stimulus and so on). The gene sets obtained through the various mechanisms outlined above when they comprised genes not in Affymetrix Probeset ID format were then converted to collections of Probeset IDs through various platforms (The Affymetrix website, matchminer, Onto tools from the Intelligent systems Bioinformatics Laboratory). Some of the gene sets that were obtained were derived from clusters obtained through classifying gene expression datasets. Sometimes, the clusters were annotated by the publications as being enriched for certain pathways or GO categories. When they were not (as for example the 'c' clusters in Mootha's published gene set list [25]), we used EASE (a tool for obtaining enrichment information on groups of genes) as we explain next. A total of 173 gene sets were constructed.

### EASE

For calculation of GO category enrichment, we used EASE. EASE is a program that determines the over-represented GO categories in a group of genes (specified either by probeset ID, accession number or other identifier) by calculating the hypergeometric ratio of the GO annotations of the genes found in an analysis versus the background distribution of each of the GO annotations. It should be noted that EASE is distinct from the various enrichment tests that are used in this paper. Enrichment techniques (such as AE, GSEA up or down regulated enrichment) measure differential expression of a set or group of genes. These groups of genes are pre-defined by us independently of the data set. EASE is independent of the enrichment techniques that we use. We can run EASE on any (possibly random) group of genes. EASE will then find what annotations are over-represented in these groups. Enrichment techniques try to see which pre-defined gene sets are most important in a re-ordered data set (thus the gene sets are previously defined and then compared to the data set), while EASE takes any group of genes and tries to find which annotations are over-represented in that group. Through the use of the various enrichment techniques, we found the hypoxia gene set to be the most significantly differentially expressed. We next ran a co-expression network analysis on this gene set.

### Co-expression network analysis

To understand connection hubs in the hypoxia pathway, a co-expression network was constructed similar to the methodology presented by Zhang and Horvath [27]. The stretch to control expression value ratios were obtained at each time point. Correlations were then obtained for all possible pairs of genes across all the time point ratios. Next we took a threshold of 0.5 of the absolute values of the correlations leaving us with correlations between genes that were higher than 0.5. The genes were then 'connected' to other genes when their pairwise correlations were above this threshold.

## Appendix

### Paired t-statistic

The paired t-statistic is given by,

$$t=(\bar{Y}-\bar{X})\sqrt{\frac{n(n-1)}{\sum_{i=1}^{n}{\left({\overset{⌢}{Y}}_{i}-{\overset{⌢}{X}}_{i}\right)}^{2}}}$$

where,

$${\overset{⌢}{X}}_{i}=(X_{i}-\bar{X}),$$

$${\overset{⌢}{Y}}_{i}=(Y_{i}-\bar{Y}),$$

$$\bar{X}=\frac{\sum_{i=1}^{n}X_{i}}{n},$$

$$\bar{Y}=\frac{\sum_{i=1}^{n}Y_{i}}{n}$$

The 'Y' variable here stands for the stretch condition while the 'X' variable stands for the control condition. We calculate the paired t-statistic for each gene ('n' is eight, the number of time points).

## Authors' contributions

VS performed the data analysis and the animal experiments. He also wrote the paper. DO provided the animals and lab facilities, insights and suggestions. IK provided the gene chips, insights and suggestions. All authors read and approved the final manuscript.
